# The adhesion molecule ICAM-1 in diffuse large B-cell lymphoma post-rituximab era: relationship with prognostic importance and rituximab resistance

**DOI:** 10.18632/aging.202180

**Published:** 2020-12-03

**Authors:** Yizhen Liu, Juan J. Gu, Ling Yang, Ping-Chiao Tsai, Ye Guo, Kai Xue, Zuguang Xia, Xiaojian Liu, Fangfang Lv, Junning Cao, Xiaonan Hong, Cory Mavis, Francisco J. Hernandez-Ilizaliturri, Qunling Zhang

**Affiliations:** 1 Department of Medical Oncology, Fudan University Shanghai Cancer Center, Department of Oncology, Shanghai Medical College, Fudan University, Shanghai, China; 2 Department of Medicine, Roswell Park Cancer Institute, Buffalo, NY 14203, USA; 3 Department of Immunology, Roswell Park Cancer Institute, Buffalo, NY 14203, USA; 4 Department of Cellular and Genetic Medicine, School of Basic Medical Sciences, Fudan University, Shanghai, China; 5 Department of Medical Oncology, Shanghai East Hospital, Tongji University School of Medicine, Shanghai, China

**Keywords:** intercellular adhesion molecule-1, diffuse large B-cell lymphoma, rituximab, chemotherapy, prognosis

## Abstract

Intercellular adhesion molecule-1 (ICAM-1) is a cell-surface receptor contributing to lymphocyte homing, adhesion and activation. The prognostic significance of the protein is unknown in diffuse large B-cell lymphoma (DLBCL) in post-rituximab era. We detected expression of ICAM-1 immunohistochemically in 102 DLBCL tissue samples. Overexpression of ICAM-1 was found in 28 (27.5%) cases. In patients with low ICAM-1 expression levels, the addition of rituximab to CHOP (cyclophosphamide, doxorubicin, vincristine and prednisone) chemotherapy resulted in an improved overall response rate, progression-free survival (PFS) and overall survival (OS) (*P*=0.019, 0.01, 0.02). In pre-clinical models, we found that chronic exposure of cell lines to rituximab led to downregulation of ICAM-1 and acquirement of a rituximab resistant phenotype. In vitro exposure of rituximab resulted in rapid aggregation of B-cells regardless of the ICAM-1 expression levels. MTT assay showed knockdown of ICAM-1 could cause rituximab resistance. Neutralization of ICAM-1 did not affect rituximab activity in vitro and in vivo. Our data illustrated that in post-rituximab era, R-CHOP significantly improved the ORR, PFS and OS in ICAM-1 negative subset patients. Downregulation of ICAM-1 may contribute to rituximab resistance, and that rituximab, by promoting cell-cell aggregation, may sensitize cells to the cytotoxic effects of chemotherapy agents.

## INTRODUCTION

Lymphoma is the fifth most common cancer in China, among which diffuse large B-cell lymphoma (DLBCL) is the predominant subtype [1]. The immunochemotherapy of rituximab combined with cyclophosphamide, doxorubicin, vincristine and prednisone (R-CHOP) significantly improved the survival rate of DLBCL patients over the last several decades [2]. However, depending on risk profile of the patient, such as International Prognostic Index (IPI), cell of origin (germinal center B-cell (GCB) or activated B-cell (ABC) subtype), and double- or triple-hit or double expression of BCL-2 and c-MYC, one third of patients will get refractory/relapse after initial treatment [3, 4]. Patients who failed initial treatment have a dismal outcome [5]. Therefore, it is necessary to investigate predictive biomarkers of response and overall survival rate in the rituximab era to establish effective target therapy.

Intercellular adhesion molecule-1 (ICAM-1) is a transmembrane glycoprotein of the immunoglobulin-like superfamily of adhesion molecules, which is constitutively expressed on lymphocytes and endothelial cells [6–8]. It contains five extracellular Ig-like domains, a transmembrane domain and a short cytoplasmic tail [9]. Binding to its ligand leukocyte function-association antigen-1(LFA-1, composed of CD18/CD11a), ICAM-1 has important contribution in lymphocyte homing, adhesion and activation. ICAM-1 is also involved in the process of tumor immune response [10–13]. Other than that, ICAM-1 was found highly expressed in solid tumors, such as liver cancer, bladder cancer, melanoma, head and neck cancer and hematologic malignancies [14–18]. In lymphoma patients before rituximab era, several papers reported that ICAM-1 expression was found correlated to the lymphoma dissemination and had prognostic value for the treatment and survival. Lymphoma patients with higher ICAM-1 expression had higher overall survival rate [19–22].

Thus, ICAM-1 seems a potential predictor of clinical outcome in pre-rituximab era. However, in rituximab era, the prognostic value of ICAM-1 has not been known in lymphoma. Here we studied the expression level of ICAM-1 in 102 diffuse large B-cell lymphoma patients and investigated its association with rituximab treated progression-free survival (PFS) and overall survival (OS). We also used rituximab-sensitive and resistant cell line models to further explore the mechanism of action.

## RESULTS

### Demographic and baseline patient characteristics

Among 102 patients who were all hospitalized, median age was 52 years (range, 19 to 72 years) at the time of diagnosis and 52 (51%) were male. According to Ann Arbor staging criteria, 67 patients (65.7%) had stage I/II diseases, and 35 patients (34.3%) had stage III/IV diseases. 80 patients (78.4%) were IPI 0 or 1 and 22 patients (21.6%) were IPI 2 or more. Eastern Cooperative Oncology Group (ECOG) scores of patients were 0-1. Concerning of cell of origin, 41 patients were germinal center B-cell lymphoma (GCB) and 56 patients were non-germinal center B-cell lymphoma (non-GCB) according to Han’s algorithm. 59 patients (57.8%) received R-CHOP and 43 patients (42.2%) received CHOP regimen as the first-line treatment. Basic features of the patients are recorded concerning the clinical and pathological parameters (Table 1).

**Table 1:** Initial characteristics and ICAM-1 expression in 102 DLBCL patients.

	**n**	**ICAM-1 Expression**		***P*-value**
		**negative (%)**	**positive (%)**	
Gender				
Female	50	37 (74.0)	13(26.0)	0.747
Male	52	37 (71.2)	15(28.8)	
Age, years				
Median	52			
≤60	73	52(71.2)	21(28.8)	0.637
>60	29	22(75.9)	7(24.1)	
ECOG score				
0	72	53(73.6)	19(26.4)	0.71
1	30	21(70.0)	9(30.0)	
Ann Arbor Stage				
I	32	19(59.4)	13(40.6)	0.232
II	35	28(80.0)	7(20.0)	
III	24	19(79.2)	5(20.8)	
IV	11	8(72.7)	3(27.3)	
Number of extranodal sites				
0	62	46(74.2)	16(25.8)	0.873
1	29	20(69.0)	9(31.0)	
>1	11	8(72.7)	3(27.3)	
LDH level				
Normal (≤250)	71	50(70.4)	21(29.6)	0.466
>Normal	31	24(77.4)	7(22.6)	
IPI score				
0-1	80	58(72.5)	22(27.5)	0.792
2	16	11(68.8)	5(31.2)	
3	6	5(83.3)	1(16.7)	
B symptom				
yes	22	15(68.2)	7(31.8)	0.604
no	80	59(73.8)	21(26.2)	
Combined with rituximab				
yes	59	40(67.8)	19(32.2)	0.208
no	43	34(79.1)	9(20.9)	
Bulky disease				
yes	43	30(69.8)	13(30.2)	0.591
no	59	44(74.6)	15(25.4)	
Cell of Origen (n=97)				
GCB	41	24(58.5)	17(41.5)	0.019
non-GCB	56	45(80.4)	11(19.6)	

### ICAM-1 expression in lymphoma tissue samples

**Figure 1:**
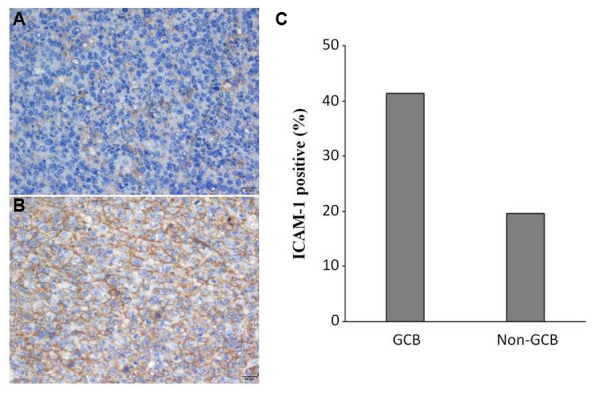
**ICAM-1 expression in DLBCL patients.** Representative immunohistochemical staining of ICAM-1 with negative (**A**) and positive (**B**) expression. (**C**) Overexpression of ICAM-1 in GCB and non-GCB subtypes.

ICAM-1 expression was observed on lymphoma cell membrane. The protein highly expressed in 28 (27.5%) cases. Image Figure 1A, 1B represented negative and positive expression of ICAM-1. Overexpression of ICAM-1 was observed more frequently in patients with GCB subtype than non-GCB subtype (41.5% vs. 19.6%, *P*=0.019) (Figure 1C).

### The association of ICAM-1 expression with ORR, PFS, and OS in CHOP and R-CHOP treated groups

**Figure 2:**
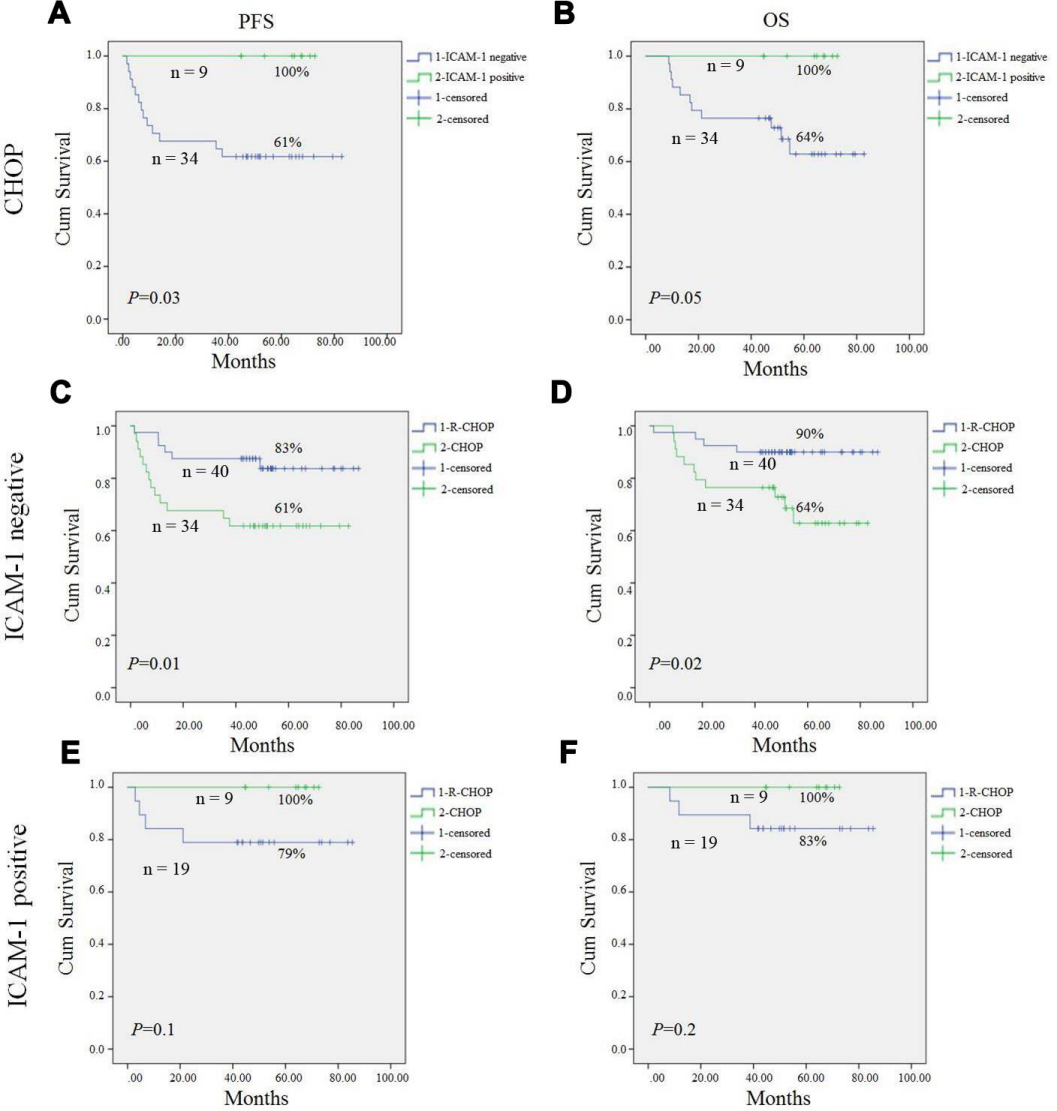
**Progression-free survival (PFS) and overall survival (OS) of DLBCL patients in our study.** Kaplan-Meier curves showing the association between ICAM-1 expression and PFS (**A**), OS (**B**) of DLBCL patients treated with CHOP regimen. R-CHOP could significantly improve PFS (**C**) and OS (**D**) in ICAM-1 negative expression patient compared to CHOP patients. In ICAM-1 positive patient, no statistical difference of PFS (**E**) and OS (**F**) between R-CHOP and CHOP group. All the *P* values are shown in the graph, by log-rank test.

Previous studies reported that lower ICAM-1 expression correlated with inferior prognosis in non-Hodgkin's lymphoma before rituximab era [19]. Our study showed results consistent with previous studies, that CHOP patients with lower expression of ICAM-1 had a worse PFS (61% vs. 100%, *P* = 0.03), and a worse OS (64% vs. 100%, *P*=0.05) compared to those with higher expression of ICAM-1 by Kaplan-Meier analysis (Figure 2A, 2B).

To our surprise, in post-rituximab era, R-CHOP significantly improved PFS and OS in ICAM-1 negative expression patients compared to CHOP patients (*P*=0.01, *P*=0.02; respectively) (Figure 2C, 2D). However, in the ICAM-1 positive patient, there was no statistical difference of PFS and OS between the two groups (Figure 2E, 2F).

As for overall response rate, in ICAM-1 positive group, there was no statistical difference between patients treated with CHOP or R-CHOP regimen (77.7% vs. 89.4%, *P*=0.409); however, in ICAM-1 negative group, an improved ORR was found in patients treated with R-CHOP compared to those with CHOP regimen (92.5% vs. 73.5%, *P*=0.019) (Table 2).

**Table 2:** Response rate of patients with different ICAM-1 expression treated by CHOP or R-CHOP regimen.

**ICAM-1**		**CR+PR**	**SD+PD**	**Total**	**ORR (%)**	***P* Value**
Positive	CHOP	7	2	9	77.7	0.409
	R-CHOP	17	2	19	89.4	
Negative	CHOP	25	9	34	73.5	0.019
	R-CHOP	37	3	40	92.5	

### Downregulation of ICAM-1 and its correlation to aggregation and anti-CD20 antibody activity in rituximab sensitive and resistant cell lines (RSCL and RRCL)

To investigate the mechanism underlying that low ICAM-1 expression group had better ORR, PFS and OS treated by R-CHOP compared to CHOP regimen, we used RSCL and RRCL generated in our lab as previous described.

**Figure 3:**
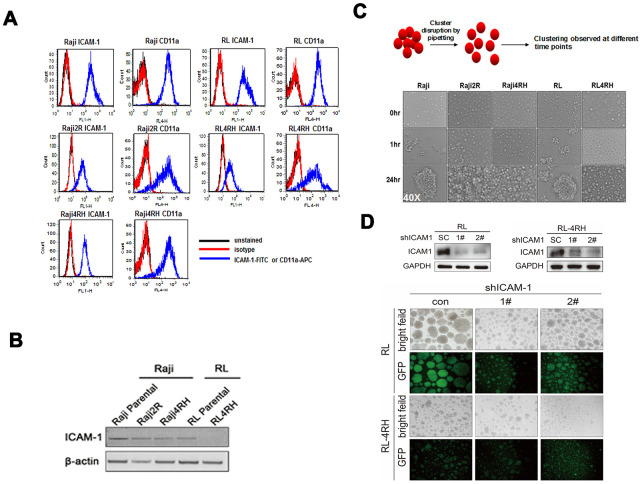
**Level of ICAM-1 and its correlation with aggregation in rituximab sensitive and resistant cell lines.** (**A**) Levels of ICAM-1 and its binding ligand CD11a in rituximab sensitive and resistant cell lines by flow cytometry staining. (**B**) Western blot showed lower expression levels of ICAM-1 in rituximab resistant cell lines (RRCL) than in rituximab sensitive cell lines (RSCL). (**C**) Ability of aggregate as cluster in RRCL and RSCL at different time points. (**D**) Knockdown of ICAM-1 by shRNA and the ability of aggregation in RSCL and RRCL.

We found the level of surface ICAM-1 protein decreased in RRCL by flow cytometry staining. However, ICAM-1 binding ligand CD11a did not have significant decrease level (Figure 3A). Western blot also validated the lower expression levels of ICAM-1 in RRCL than in RSCL (Figure 3B). Since ICAM-1 is the major adhesion molecule, further investigation revealed that RRCL did not aggregate as a cluster together as RSCL did (Figure 3C). We also used shRNA to knockdown ICAM-1 in RSCL and RRCL, and found that cell clustering is dependent on the ICAM-1 expression levels (Figure 3D). Anti-CD20 antibodies (rituximab, ofatumumab, TG20, R603 and GA101) dependent cellular toxicity (ADCC) and complement dependent cytotoxicity (CMC) were all decreased in rituximab resistant cells compared to its parental sensitive cell lines (Table 3). All these suggested decreased ICAM-1 expression correlated with loss of cellular aggregation and decreased CD20 antibody activity in RRCL.

**Table 3:** ADCC and CMC assays detecting anti-CD20 antibody activity in RRCL and RSCL.

**Cell Line**	**% ADCC (N=3)**					**% CMC (N=4)**				
	**R**	**Ofatumumab**	**TG20**	**R603**	**GA101**	**R**	**Ofatumumab**	**TG20**	**R603**	**GA101**
Raji	25.99	30.82	47.9	53.57	58	79.87	87	82.05	80.08	12.87
Raji2R	14.35	14.63	26.84	29.78	39.41	3.26	8.68	4.6	1.94	0.89
Raji4RH	7.03	6.88	13.2	15.36	23.56	3.62	9.42	4.4	3.72	2.45
RL	22.62	25.9	45.65	52.1	56.45	89.52	91.28	88.86	88.94	13.98
RL4RH	17.07	17.88	29.72	32.78	42.07	4.54	9.07	7.11	4.64	3.77

### Rituximab enhances cellular aggregation in both ICAM-1 high expressed RSCL and ICAM-1 low expressed RRCL

**Figure 4:**
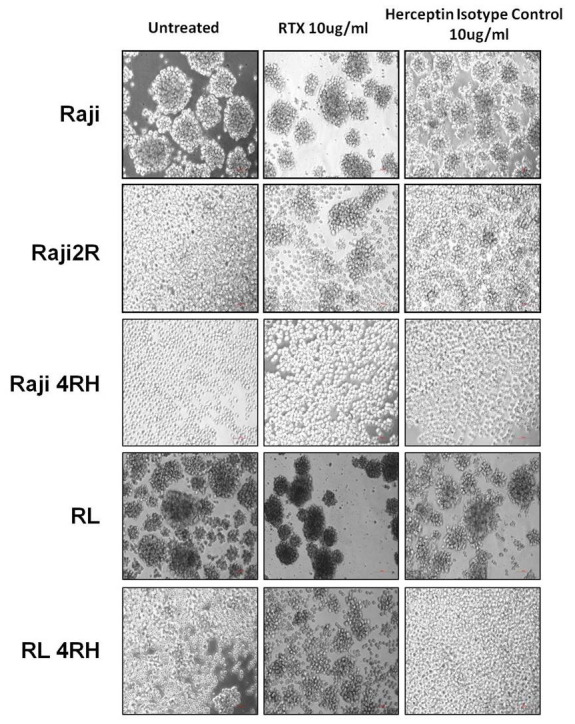
**Cellular aggregation in RSCL and RRCL treated with rituximab.** Rituximab enhances cellular aggregation in both ICAM-1 high expressed RSCL and ICAM-1 low expressed RRCL.

The major cellular killing activity of rituximab is by CMC and ADCC, both of which depend on cellular interaction with complement and/or other cells. We observed that both RSCL and RRCL aggregated after rituximab treatment, regardless of ICAM-1 expression levels (Figure 4). Furthermore, We performed western blot to examine expression levels of other adhesion molecules (ICAM-3, VCAM-1 and E-cad) in RSCL, and found all of them showed low or no expression (data not shown).

### Knockdown of ICAM-1 causes rituximab resistance

**Figure 5:**
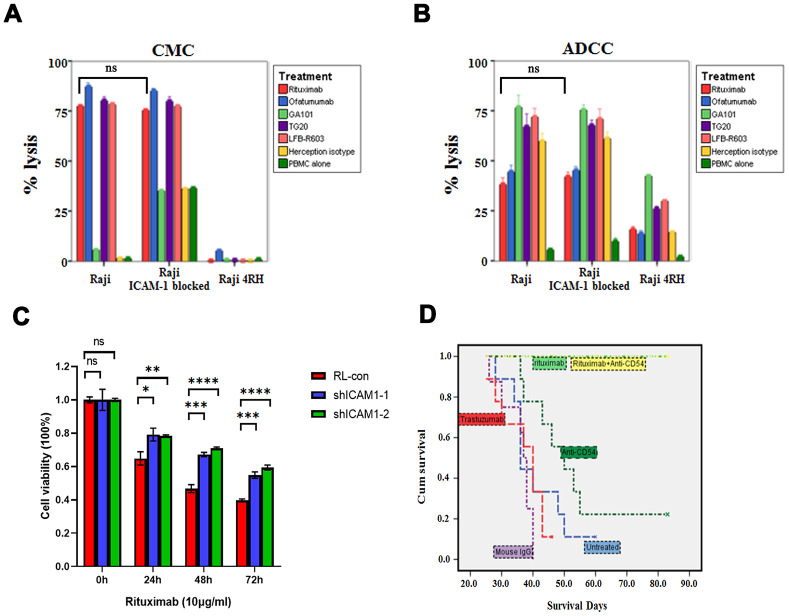
**Impacts of neutralization of ICAM-1 in vitro and in vivo.** (**A, B**) Neutralization of ICAM-1 did not affect rituximab and other anti-CD20 antibodies mediated CMC and ADCC in rituximab sensitive cell lines. (**C**) MTT assay showed that downregulation of ICAM-1 could cause resistance of rituximab. (**D**) Kaplan-Meier analyses showing the survival curves of neutralization of ICAM-1 alone and in combination with rituximab in vivo. (*P<0.05, **P <0.01, ***P <0.001,****P <0.0001).

We neutralized ICAM-1 in Raji cell line, which had high expression of ICAM-1, and found there were no statistical differences of rituximab mediated CMC and ADCC between the control group and ICAM-1 neutralization group in vitro (*P*> 0.05, Figure 5A, 5B). However, MTT assay showed that downregulation of ICAM-1 could cause resistance of rituximab (*P* values are shown in Figure 5C). In vivo, rituximab combined with ICAM-1 neutralization did not affect rituximab killing activity (*P*> 0.05) (Figure 5D).

## DISCUSSION

DLBCL is a heterogeneous disease in both clinical and biological settings. Many factors identified have prognostic values, such as Ki67, BCL-2 and c-MYC etc [23, 24]. ICAM-1 is an adhesion molecule normally expressed on the surface of lymphocytes and endothelial cells. It was also found expressed on various cancer cells, including head and neck cancer, melanoma and lymphoma. Previous study showed that absence or low expression of ICAM-1 was correlated with bone marrow infiltration, advanced stage and dismal clinical outcome in pre-rituximab era [19–22, 25].

In our study, ICAM-1 was detected mainly on the membrane. In pre-rituximab era, Kaplan-Meier analysis indicated that patients with high expression of ICAM-1 had a better PFS compared to those with low expression of the protein in the CHOP group (*P*=0.03). OS between the two group showed a strong tendency towards statistical significance (*P* =0.05), which is consistent with other reports [19, 21, 22]. But in rituximab era, to our surprise, we found there were no significant differences in PFS and OS between the ICAM-1 negative patient and positive patients.

Furthermore, we found that in ICAM-1 negative patients, R-CHOP regimens achieved a significant higher ORR, PFS and OS than CHOP regimens. However, in ICAM-1 positive patients, no difference was found. This indicated ICAM-1 negative patient may gain more benefit from rituximab combined treatment than the ICAM-1 positive patients.

Although higher ICAM-1 was detected in GCB subtype patients (*P*=0.01), the association between patients with GCB or non-GCB and their PFS and OS was not found statistical difference.

Considering molecular mechanism, correlation between ICAM-1 expression, cellular aggregation and rituximab anti-tumor activity was determined by flow cytometry, aggregation assay, CMC and ADCC assays as well as MTT assay. Loss of ICAM-1 expression level was found in rituximab resistant cell lines, along with reduction of cellular aggregation and rituximab activity. The major cellular killing activity of rituximab is by ADCC and CMC, both of which depend on cellular interaction with complement and/or other cells. Previously studies also revealed that rituximab could modify intracellular targets, and could sensitize cells to the cytotoxic effects of chemotherapy agents [26, 27]. We and others found that rituximab increased cellular aggregation independent of ICAM-1 expression level [28–30]. It is possible that rituximab may partially compensate the loss of cellular adhesion caused by low ICAM-1 level in patients treated with R-CHOP. In rituximab sensitive cell lines which ICAM-1 is relatively over-expressed, we found that knockdown of ICAM-1 could cause resistance of rituximab. However, neutralization of ICAM-1 did not affect rituximab-mediated ADCC and CMC activity in vitro and in vivo. The possible reason might be that shRNA could integrated into the genome for gene silencing, while neutralizing antibodies would not block the surface proteins entirely. Moreover, rituximab had other anti-tumor activities besides ADCC and CMC [31]. These may also explain that rituximab-based treatment had good PFS and OS independent of ICAM-1 expression levels in the clinical setting.

Our clinical data suggested that patients with low ICAM-1 expression level identified before treatment had superior outcome when receiving R-CHOP regimen. However, exposure to rituximab caused loss of ICAM-1 and acquired resistance. The contribution of the loss of ICAM-1 to drug-resistance remains utterly unknown and needs to be further investigated.

Overall, our research found ICAM-1 expression level was higher in GCB than non-GCB subtype. Before the rituximab era, ICAM-1 low expression correlated with worse PFS. This is the first time to report that rituximab significantly improved the ORR, PFS and OS in ICAM-1 negative subset patients. Loss of ICAM-1 after exposed lymphoma cell to rituximab may contribute to the resistance to rituximab.

## MATERIALS AND METHODS

### Patients and samples

A total of 102 patients were included in this retrospective study that were newly diagnosed of DLBCL and treated in Fudan University Shanghai Cancer Center, Shanghai, China from March 2009 to December 2012. All patients were pathologically confirmed as DLBCL through surgical or biopsy samples, and all pathological results were reviewed by experienced pathologists in pathology department of Fudan University Shanghai Cancer Center. The demographic details, clinical and laboratory features of the patients are summarized in Table 1. This study was approved by the Institutional Review Board of the Fudan University Shanghai Cancer Center. Survival data were available with a median follow-up of 1,545 days (range 45~2,598 days).

### Immunohistochemistry (IHC)

Immunohistochemical staining was carried out with the Dako Envision System (Dako, Glostrup, Denmark). In brief, formalin-fixed, paraffin-embedded tissue sections were incubated overnight at 4° C with mouse monoclonal antibody against ICAM-1 (Santa Cruz, CA, USA) at 1:100 dilutions. Slides with no primary antibodies added served as negative controls.

### Immunohistochemical assessment

The expression level of ICAM-1 was assessed by percentage of positive cells. Tumors were considered positive when at least 75% of tumor cells expressed ICAM-1. Assessment was done by three pathologists without prior knowledge of the clinical features or follow-up data of the patients.

### Cell lines

Human lymphoma cell line Raji and RL were purchased from American Type Culture Collection (ATCC, Manassas, VA). Rituximab resistant cell line Raji 4RH, RL 4RH were generated by exposure sensitive cells to rituximab at escalating dosages over times in the presence of human complement as previously described [32].

All the cell lines were maintained in RPMI 1640 supplemented with 10% heat-inactivated fetal bovine serum, 100 U/ml penicillin and 100 g/ml streptomycin, maintained in 5% CO2 at 37° C.

### Disruption of cell cluster aggregation for spontaneous re-aggregation study

Disruption of rituximab sensitive cell line (RSCL) clusters at the concentration of 1×10^6^ cells/ml were achieved by gentle pipetting before plating on a 6-well plate. Re-aggregation of cell cluster was then observed, and pictures were taken under microscope at different time intervals.

### Effects of rituximab on spontaneous cell cluster aggregation

1×10^6^ cells were centrifuged and RPMI 1640-10%FBS media was removed, leaving 500ul in the tube with cells. Calculated volume of rituximab, or isotype controls was added to each tube of cells. Cells were resuspended briefly so that they mixed well with antibody, and then incubated for 1hr in 37° C incubator for binding of antibody to cell surface to occur. After 1hr incubation, 1ml of RPMI1640-10% FBS media was added back to each tube. Cells were again resuspended well by gentle pipetting before being transferred to a 6-well plate.

### Flow cytometry for ICAM-1 detection

RSCL and rituximab resistant cell lines (RRCL) were treated with rituximab or Herceptin isotype control for 48h. Then the cells were stained with either FITC-conjugated mouse anti-human ICAM-1 (BD Invitrogen) or Isotype control (mouse IgG2α-FITC) (BD Invitrogen) for 30 minutes. Cells were then washed with PBS, fixed with 2% paraformaldehyde and analyzed by flow cytometry using FACSCCalibur (BD Biosciences). Cells were analyzed using the FCS express software (De Novo Software, Los Angeles, CA, USA).

### Western blot

Cells were lysed in 200 μl lysis buffer (12.5mM Tris-HCL PH 6.8, 4% SDS, 20% glycerol, 0.004% bromophenol blue). The protein extracts (20 μg) were separated by 10% SDS–PAGE and transferred to polyvinylidene fluoride membranes (Millipore, Temecula, CA, USA). The membrane was blocked for 1 hour by 5% non-fat milk and then incubated with primary antibodies overnight at 4 ° C. After washed 3 times with Tris-buffered saline/0.1% Tween 20, the membrane was incubated with horseradish peroxidase-linked secondary antibodies for 1 hour. Proteins were visualized using Tanon full-automatic light detecting system with the BeyoECL Star (Ultra hypersensitive ECL chemiluminescence kit). All the data were confirmed by three individual experiments.

### Plasmid, lentivirus production and infection

The forward sequences of shRNAs targeting ICAM1 were as follows: sh-1: CCGGCCGGTATGAGATTGTCATCATCTCGAGATGATGACAATCTCATACCGGTTTTTG;sh-2:CCGGGCCAACCAATGTGCTATTCAACTCGAGTTGAATAGCACATTGGTTGGCTTTTTG. The shRNA oligo sequences were cloned into a lentiviral shRNA expressing plasmid GV248 (GeneChem, Shanghai). A scramble sequence TTCTCCGAACGTGTCACGT constructed as stem-loop-stem structure were also cloned into the same plasmid and were used as control (SC). For the production of lentivirus, 293T cells were transfected with shRNA-expressing plasmids (SC, shICAM1-1, or shICAM1-2) and packaging plasmids (PSPAX2 and pMD2G) by Lipofectamine® 3000 (Invitrogen). Virus supernatants were harvested at 48h, 72h and 96h after transfection and were used to infect cultured cell lines. The infected cells were then isolated by puromycin selection.

### Antibody-dependent cellular cytotoxicity (ADCC) and complement-mediated cytotoxicity (CMC)

The ADCC and CDC were accessed by ^51^Cr release assay. In brief, cells were labeled with ^51^Cr at 37° C, 5% CO_2_ for 2 hours and then were plated at a cell concentration of 1x10^5^ cells/well (CMC assay) or 1x10^4^ cells/well (ADCC assay). Cells were then exposed to rituximab (10μg/ml), ofatumumab, TG20, R603 and GA101 or isotype (10μg/ml) and human serum (CMC, 1:4 dilution) or PBMCs (ADCC, 40:1 effector: target ratio) for six hours at 37° C and 5% CO_2_. ^51^Cr release was measured and percentage of cell-lysis was calculated as previously described.

### Cell viability assay (MTT assay)

RSCL was plated at a cell density of 1 × 10^5^ cells/ml in 96 well plates and was exposed to 10ug/ml of rituximab for different time intervals. At the indicated time points, 20 μl of 0.5 mg/ml MTT (Thiazolyl Blue Tetrazolium Bromide, Sigma) was added to each well and incubated at 37° C for 4 h. In the end, the culture was replaced with 150 μL DMSO (dimethyl sulfoxide, D8418, Sigma) to stop the reaction. The absorbance values (OD 570 nm) were measured using a spectrophotometer.

### Analyses of the effect of ICAM-1 neutralization in RSCL on tumor growth in SCID mouse model

Following 1hr incubation with the indicated antibodies (10μg/ml rituximab, 0.25 μg/ml ICAM-1 neutralizing antibody (Calbiochem), or isotype controls), Raji cells were inoculated into SCID mice through intraperitoneally. The growth of tumor was measured twice per week and mice were sacrificed when one dimension of the tumor reached 2cm, or when necrotic/ulcerated tumor developed. The average cumulative days of survival were calculated.

### Statistical analysis

The analyses were performed using SPSS 23.0 (SPSS Inc., Chicago) or GraphPad Prism 8 software (GraphPad Software, Inc., La Jolla, CA). Associations between protein expression and clinical/laboratory parameters or treatment response were assessed by χ^2^ test. Differences of the cell viability between control and sh-ICAM-1 cell lines treated with rituximab were analyzed using students t-test. Kaplan-Meier survival curves were constructed for survival analyses, and differences were tested by the log-rank test. OS was defined as the time between the date of surgery or biopsy and the date of death or the date of last contact. PFS refers to the period from the beginning of treatment to the observed progression of the disease or the occurrence of death for any reason. The data of patients alive at the end of the study were censored. All *P* values were two-sided, and the results were considered significant if *P* < 0.05.

## References

[1] Kubuschok B, Held G, Pfreundschuh M. Management of diffuse large B-cell lymphoma (DLBCL). Cancer Treat Res. 2015; 165:271–88. 10.1007/978-3-319-13150-4_11 25655614

[2] Friedberg JW. Relapsed/refractory diffuse large B-cell lymphoma. Hematology Am Soc Hematol Educ Program. 2011; 2011:498–505. 10.1182/asheducation-2011.1.498 22160081

[3] International Non-Hodgkin's Lymphoma Prognostic Factors Project. A predictive model for aggressive non-Hodgkin's lymphoma. N Engl J Med. 1993; 329:987–94. 10.1056/NEJM199309303291402 8141877

[4] Sehn LH, Berry B, Chhanabhai M, Fitzgerald C, Gill K, Hoskins P, Klasa R, Savage KJ, Shenkier T, Sutherland J, Gascoyne RD, Connors JM. The revised International Prognostic Index (R-IPI) is a better predictor of outcome than the standard IPI for patients with diffuse large B-cell lymphoma treated with R-CHOP. Blood. 2007; 109:1857–61. 10.1182/blood-2006-08-038257 17105812

[5] Kochenderfer JN, Rosenberg SA. Treating b-cell cancer with T cells expressing anti-CD19 chimeric antigen receptors. Nat Rev Clin Oncol. 2013; 10:267–76. 10.1038/nrclinonc.2013.46 23546520 PMC6322669

[6] Makgoba MW, Sanders ME, Ginther Luce GE, Dustin ML, Springer TA, Clark EA, Mannoni P, Shaw S. ICAM-1 a ligand for LFA-1-dependent adhesion of B, T and myeloid cells. Nature. 1988; 331:86–88. 10.1038/331086a0 3277059

[7] Smith ME, Thomas JA. Cellular expression of lymphocyte function associated antigens and the intercellular adhesion molecule-1 in normal tissue. J Clin Pathol. 1990; 43:893–900. 10.1136/jcp.43.11.893 1702102 PMC502897

[8] Maio M, Del Vecchio L. Expression and functional role of CD54/Intercellular Adhesion Molecule-1 (ICAM-1) on human blood cells. Leuk Lymphoma. 1992; 8:23–33. 10.3109/10428199209049814 1362919

[9] Momosaki S, Yano H, Ogasawara S, Higaki K, Hisaka T, Kojiro M. Expression of intercellular adhesion molecule 1 in human hepatocellular carcinoma. Hepatology. 1995; 22:1708–13. 10.1002/hep.1840220615 7489978

[10] Boyd AW, Wawryk SO, Burns GF, Fecondo JV. Intercellular adhesion molecule 1 (ICAM-1) has a central role in cell-cell contact-mediated immune mechanisms. Proc Natl Acad Sci USA. 1988; 85:3095–99. 10.1073/pnas.85.9.3095 3362863 PMC280150

[11] Wawryk SO, Novotny JR, Wicks IP, Wilkinson D, Maher D, Salvaris E, Welch K, Fecondo J, Boyd AW. The role of the LFA-1/ICAM-1 interaction in human leukocyte homing and adhesion. Immunol Rev. 1989; 108:135–61. 10.1111/j.1600-065x.1989.tb00016.x 2670740

[12] Gregory CD, Murray RJ, Edwards CF, Rickinson AB. Downregulation of cell adhesion molecules LFA-3 and ICAM-1 in Epstein-Barr virus-positive Burkitt’s lymphoma underlies tumor cell escape from virus-specific T cell surveillance. J Exp Med. 1988; 167:1811–24. 10.1084/jem.167.6.1811 2898508 PMC2189677

[13] Stauder R, Greil R, Schulz TF, Thaler J, Gattringer C, Radaskiewicz T, Dierich MP, Huber H. Expression of leucocyte function-associated antigen-1 and 7F7-antigen, an adhesion molecule related to intercellular adhesion molecule-1 (ICAM-1) in non-Hodgkin lymphomas and leukaemias: possible influence on growth pattern and leukaemic behaviour. Clin Exp Immunol. 1989; 77:234–38. 2673591 PMC1541990

[14] Sun JJ, Zhou XD, Liu YK, Tang ZY, Feng JX, Zhou G, Xue Q, Chen J. Invasion and metastasis of liver cancer: expression of intercellular adhesion molecule 1. J Cancer Res Clin Oncol. 1999; 125:28–34. 10.1007/s004320050238 10037274 PMC12199857

[15] Benedicto A, Romayor I, Arteta B. Role of liver ICAM-1 in metastasis. Oncol Lett. 2017; 14:3883–92. 10.3892/ol.2017.6700 28943897 PMC5604125

[16] Jackson AM, Alexandrov AB, Gribben SC, Esuvarnathan K, James K. Expression and shedding of ICAM-1 in bladder cancer and its immunotherapy. Int J Cancer. 1993; 55:921–25. 10.1002/ijc.2910550608 7902828

[17] Galore-Haskel G, Baruch EN, Berg AL, Barshack I, Zilinsky I, Avivi C, Besser MJ, Schachter J, Markel G. Histopathological expression analysis of intercellular adhesion molecule 1 (ICAM-1) along development and progression of human melanoma. Oncotarget. 2017; 8:99580–86. 10.18632/oncotarget.20884 29245925 PMC5725116

[18] Maio M, Pinto A, Carbone A, Zagonel V, Gloghini A, Marotta G, Cirillo D, Colombatti A, Ferrara F, Del Vecchio L. Differential expression of CD54/intercellular adhesion molecule-1 in myeloid leukemias and in lymphoproliferative disorders. Blood. 1990; 76:783–90. 10.1182/blood.V76.4.783.783 1974471

[19] Terol MJ, López-Guillermo A, Bosch F, Villamor N, Cid MC, Rozman C, Campo E, Montserrat E. Expression of the adhesion molecule ICAM-1 in non-Hodgkin’s lymphoma: relationship with tumor dissemination and prognostic importance. J Clin Oncol. 1998; 16:35–40. 10.1200/JCO.1998.16.1.35 9440720

[20] Terol MJ, Tormo M, Martinez-Climent JA, Marugan I, Benet I, Ferrandez A, Teruel A, Ferrer R, García-Conde J. Soluble intercellular adhesion molecule-1 (s-ICAM-1/s-CD54) in diffuse large B-cell lymphoma: association with clinical characteristics and outcome. Ann Oncol. 2003; 14:467–74. 10.1093/annonc/mdg057 12598355

[21] Aboul-Enein M, El-Sayed GM, El-Maghraby S, Abd-Elatif NA, Abd Elwahab GA, Elbasmy AA. Intercellular adhesion molecule-1(ICAM-1), CD44s expression and serum level of sICAM-1 in disseminated non-Hodgkin’s lymphoma: correlation with overall survival. J Egypt Natl Canc Inst. 2004; 16:244–51. 16116502

[22] Shah N, Cabanillas F, McIntyre B, Feng L, McLaughlin P, Rodriguez MA, Romaguera J, Younes A, Hagemeister FB, Kwak L, Fayad L. Prognostic value of serum CD44, intercellular adhesion molecule-1 and vascular cell adhesion molecule-1 levels in patients with indolent non-Hodgkin lymphomas. Leuk Lymphoma. 2012; 53:50–56. 10.3109/10428194.2011.616611 21895545 PMC4104165

[23] Sehn LH, Gascoyne RD. Diffuse large B-cell lymphoma: optimizing outcome in the context of clinical and biologic heterogeneity. Blood. 2015; 125:22–32. 10.1182/blood-2014-05-577189 25499448

[24] Costa LJ, Maddocks K, Epperla N, Reddy NM, Karmali R, Umyarova E, Bachanova V, Costa C, Glenn MJ, Chavez JC, Calzada O, Lansigan F, Nasheed H, et al. Diffuse large B-cell lymphoma with primary treatment failure: ultra-high risk features and benchmarking for experimental therapies. Am J Hematol. 2017; 92:161–70. 10.1002/ajh.24615 27880984 PMC5549936

[25] Ponzoni M, Arrigoni G, Gould VE, Del Curto B, Maggioni M, Scapinello A, Paolino S, Cassisa A, Patriarca C. Lack of CD 29 (beta1 integrin) and CD 54 (ICAM-1) adhesion molecules in intravascular lymphomatosis. Hum Pathol. 2000; 31:220–26. 10.1016/s0046-8177(00)80223-3 10685637

[26] Jazirehi AR, Bonavida B. Cellular and molecular signal transduction pathways modulated by rituximab (rituxan, anti-CD20 mAb) in non-Hodgkin’s lymphoma: implications in chemosensitization and therapeutic intervention. Oncogene. 2005; 24:2121–43. 10.1038/sj.onc.1208349 15789036

[27] Johnson P, Glennie M. The mechanisms of action of rituximab in the elimination of tumor cells. Semin Oncol. 2003; 30:3–8. 10.1053/sonc.2003.50025 12652458

[28] Vega MI, Huerta-Yepez S, Martinez-Paniagua M, Martinez-Miguel B, Hernandez-Pando R, González-Bonilla CR, Chinn P, Hanna N, Hariharan K, Jazirehi AR, Bonavida B. Rituximab-mediated cell signaling and chemo/immuno-sensitization of drug-resistant B-NHL is independent of its Fc functions. Clin Cancer Res. 2009; 15:6582–94. 10.1158/1078-0432.CCR-09-1234 19861448

[29] Kennedy AD, Beum PV, Solga MD, DiLillo DJ, Lindorfer MA, Hess CE, Densmore JJ, Williams ME, Taylor RP. Rituximab infusion promotes rapid complement depletion and acute CD20 loss in chronic lymphocytic leukemia. J Immunol. 2004; 172:3280–88. 10.4049/jimmunol.172.5.3280 14978136

[30] Alinari L, Yu B, Christian BA, Yan F, Shin J, Lapalombella R, Hertlein E, Lustberg ME, Quinion C, Zhang X, Lozanski G, Muthusamy N, Prætorius-Ibba M, et al. Combination anti-CD74 (milatuzumab) and anti-CD20 (rituximab) monoclonal antibody therapy has in vitro and in vivo activity in mantle cell lymphoma. Blood. 2011; 117:4530–41. 10.1182/blood-2010-08-303354 21228331 PMC3099572

[31] Weiner GJ. Rituximab: mechanism of action. Semin Hematol. 2010; 47:115–23. 10.1053/j.seminhematol.2010.01.011 20350658 PMC2848172

[32] Czuczman MS, Olejniczak S, Gowda A, Kotowski A, Binder A, Kaur H, Knight J, Starostik P, Deans J, Hernandez-Ilizaliturri FJ. Acquirement of rituximab resistance in lymphoma cell lines is associated with both global CD20 gene and protein down-regulation regulated at the pretranscriptional and posttranscriptional levels. Clin Cancer Res. 2008; 14:1561–70. 10.1158/1078-0432.CCR-07-1254 18316581

